# Cadherin-11 contributes to liver fibrosis induced by carbon tetrachloride

**DOI:** 10.1371/journal.pone.0218971

**Published:** 2019-07-03

**Authors:** Mesias Pedroza, Sarah To, Jennifer Smith, Sandeep K. Agarwal

**Affiliations:** Section of Immunology, Allergy and Rheumatology, Baylor College of Medicine, Houston, TX, United States of America; University of Navarra School of Medicine and Center for Applied Medical Research (CIMA), SPAIN

## Abstract

**Background and aims:**

Liver fibrosis is characterized by the excessive deposition of extracellular matrix (ECM) leading to impaired function and cirrhosis. Previous reports support a role for cadherin-11 (CDH11) in regulating the development of dermal and pulmonary fibrosis. In the current report, the extent to which CDH11 modulates the development of liver fibrosis induced by carbon tetrachloride (CCL_4_) was assessed.

**Methods:**

Wild type (WT) and CDH11 deficient (*CDH11*^*-/-*^) mice were treated with CCl_4_ or vehicle control for 8 weeks to induce liver fibrosis. Liver fibrosis was assessed by histology, collagen content, and RTPCR of fibrotic mediators.

**Results:**

Livers from WT mice treated with CCl_4_ had increased levels of CDH11 which localized to injured hepatocytes, hepatic stellate cells, and macrophages. Interestingly, *CDH11*^*-/-*^ mice had decreased histological evidence of liver fibrosis, collagen deposition, α-smooth muscle actin (α-SMA) accumulation, and mRNA levels of fibrotic mediators such as *Col1-*α*1*, *Snail*, *TGF-*β and *IL-6*.

**Conclusions:**

These data demonstrate that CDH11 is increased during liver fibrosis, is an important regulator of liver fibrosis induced by CCL_4_ and suggest that CDH11 may be a potential therapeutic target for liver fibrosis.

## Introduction

Chronic liver disease and cirrhosis are the 12^th^ leading cause of death in the United States, with a mortality rate of 12.5 per 100,000 people and mortality rates are on the rise [1, 2]. Liver fibrosis is a common, downstream pathological process that results from a variety noxious stimuli including medications, alcohol, viruses and autoimmunity [2]. During the development of liver fibrosis excessive extracellular matrix (ECM) disrupts the normal liver architecture leading to cirrhosis. Understanding the molecular pathogenesis of fibrosis is needed to identify novel therapeutic targets for the treatment of liver fibrosis and cirrhosis.

Fibrogenesis in the liver involves multiple cellular populations, including hepatocytes, hepatic stellate cells (HSC), resident fibroblasts, and macrophages. In response to injury, cells in the liver participate in a wound healing response seeking to restore tissue function. However, persistence of injury leads to a dysregulated reparative process, the accumulation of myofibroblasts, the deposition of ECM, and fibrosis [3]. Hepatocyte necrosis and apoptosis trigger an inflammatory response including cytokines and chemokines that recruit inflammatory cells, such as macrophages (Kupffer cells) [4]. Engulfment of hepatocyte apoptotic bodies by Kupffer cells and HSC leads to a pro-fibrotic milieu with increased transforming growth factor (TGF-β), interleukin-6 (IL-6), platelet-derived growth factor, and endothelial growth factor. These factors activate and trigger HSCs to undergo myofibroblast differentiation [4]. Other sources of myofibroblasts include activated resident fibroblasts and possibly hepatocytes that undergo epithelial–mesenchymal transition (EMT) [5, 6]. Ultimately, these myofibroblasts deposit large amounts of ECM into the liver parenchyma, leading to fibrosis. [2].

Cadherin-11 (CDH11) is a calcium-dependent, type II classical cadherin initially identified on osteoblasts, but now known to be expressed by a number of cells involved in the development of fibrosis including fibroblasts, myofibroblasts, injured type II alveolar epithelial cells, and macrophages [7–9]. Neutralization and genetic deletion of CDH11 reduced lung fibrosis in a model of bleomycin-induced pulmonary fibrosis in mice [8] and skin fibrosis in two different mouse models [7, 9]. These data have strongly implicated CDH11 as a mediator and potential therapeutic target in lung and skin fibrosis. Given common pathways involved in fibrosis in multiple organs, we hypothesized that CDH11 expression is increased during liver injury and contributes to the development of liver fibrosis induced by carbon tetrachloride (CCl_4_).

## Methods

### Mice

These studies were approved by Baylor College of Medicine Institutional Animal Care and Use Committee and animal care was in accordance with institutional and NIH guidelines. Mice were housed in ventilated cages equipped with microisolator lids, with corncob bedding, standard chow, with free access to food and water in a specific pathogen free facility. Cadherin-11 deficient mice (*CDH11*^*-/-*^) on the C129-C57/B6 background and matched wild-type (WT) mice were used in this study [10]. The experiments were done in accordance with the Animal Research: Reporting of *In vivo* Experiments (ARRIVE) guidelines (S1 File).

### Intra-peritoneal carbon tetrachloride exposure

WT and CDH11^-/-^ male mice (age 5-weeks old) were injected intraperitoneally (i.p.) with 1 μl/g CCl_4_ (Sigma-Aldrich, diluted 1:3 in corn oil) or with the vehicle (corn oil, Sigma-Aldrich) twice a week for 8 weeks. Mice were euthanized by exsanguination while under isoflurane anesthesia four days after last injection. Data was obtained from 4 individual experiments and each individual experiment was performed with 3–4 mice per group (total sample size: 14 mice per group).

### Histology

Formalin-fixed paraffin-embedded liver biopsy samples (5 μm thick sections) were stained with hematoxylin and eosin (H&E) or Masson’s Trichrome to evaluate the level of liver fibrosis. Ishak scoring of H&E-stained liver sections was used to quantify the amount of liver fibrosis as previously described [11]. Significant fibrosis was defined as Ishak score ≥ 4. Experiments were conducted with 14 mice per group and histology was scored using 20 fields per section in blinded manner.

For immunohistochemistry (IHC), tissue sections were rehydrated and antigen retrieval was performed (Dako). Endogenous peroxidases were quenched and endogenous avidin and biotin were blocked with BLOX ALL blocking solution (Vector Labs). Slides were blocked with 2.5% horse serum (Vector Labs). Slides were incubated over night at 4 degrees Celsius with primary antibodies for α-SMA (1:1000 dilution, Sigma-Aldrich) or mouse IgG and processed with the Mouse on Mouse Kit and the ImmPACT Vector Red substrate kit (Vector Labs).

For immunofluorescence (IF) formalin-fixed, paraffin-embedded sections were [12]. After blocking, slides were incubated at 4 degrees Celsius overnight with Alexa Fluor 488-conjugated antibody against CDH11 (R&D systems) and antibodies against α-SMA (Sigma-Aldrich), α1-Fetoprotein (Abcam, ab114028), Synaptophysin (Abcam, ab8049), or F4/80 (1:1000 dilution, Abcam, ab100790). Isotype controls include mouse or rabbit IgG. Secondary antibodies were conjugated with Alexa flour 647. Sections were mounted with ProLong Gold antifade reagent with DAPI (Life Technologies).

### Whole-liver RNA analysis

Total RNA was isolated from mouse livers utilizing Trizol (Invitrogen) and used for quantitative real-time RT-PCR analysis using validated TaqMan Gene Expression Assays for CDH11, Col1-α1, α-SMA, Snail, TGF-β, CCN2, IL-6, TIMP1, MMP13, MMP3, Lumican, and 18s rRNA (Applied Biosystems) on an Applied Biosystems Step One Plus PCR System. The 18s rRNA gene was used as an endogenous control to normalize transcript levels of mRNA in each sample and presented as mean normalized transcript levels using the comparative Ct method (2^ΔΔCt^).

### Biochemical analysis of liver biopsies

Collagen content was determined by Sircol Collagen Assay kit (Biocolor, Newtown Abbey, UK). Total protein assay (Bio-Rad Laboratories, Hercules, CA) was used as control to normalize collagen content of each sample. Alanine transaminase (ALT) activity was determined in serum samples using a commercial colorimetric assay (Abcam, ab105134).

### Cell culture

LX2, a human hepatic stellate cell line, and AML-12, a murine hepatocyte cell line, were obtained from Dr. David Moore at BCM. Cells were cultured in DMEM medium containing 5% FBS and 1% penicillin/streptomycin. For experiments, cells were stimulated with: media alone or TGF-β (10 ng/ml) (R&D Systems, 240-B-002) for 24 hours. Cells were harvested at 24 hours post-treatment for RNA analysis using TaqMan Gene Expression Cells-to-CT Kit (Thermo Fisher Scientific, AM1729). Real-time PCR was performed on an Applied Biosystems Step One Plus PCR System using validated TaqMan Gene Expression Assays for CDH11, Col1-α1, α-SMA, Snail, TGF-β, N-Cad, and 18s rRNA (Applied Biosystems). Transcript levels of mRNA in each sample were normalized using the 18s rRNA gene as an endogenous control. Data with each cell type were obtained from 4 individual experiments in triplicates and is presented as mean normalized transcript levels using the comparative Ct method (2ΔΔCt).

### Statistics

Values are expressed as mean ± SEM. As appropriate, WT and CDH11^-/-^ mice were compared by analysis of variance and 2-tailed Student’s t test. A p-value of ≤ 0.05 was considered to be significant.

## Results

### Increased CDH11 expression in CCL_4_ mouse model of liver fibrosis

**Fig 1:**
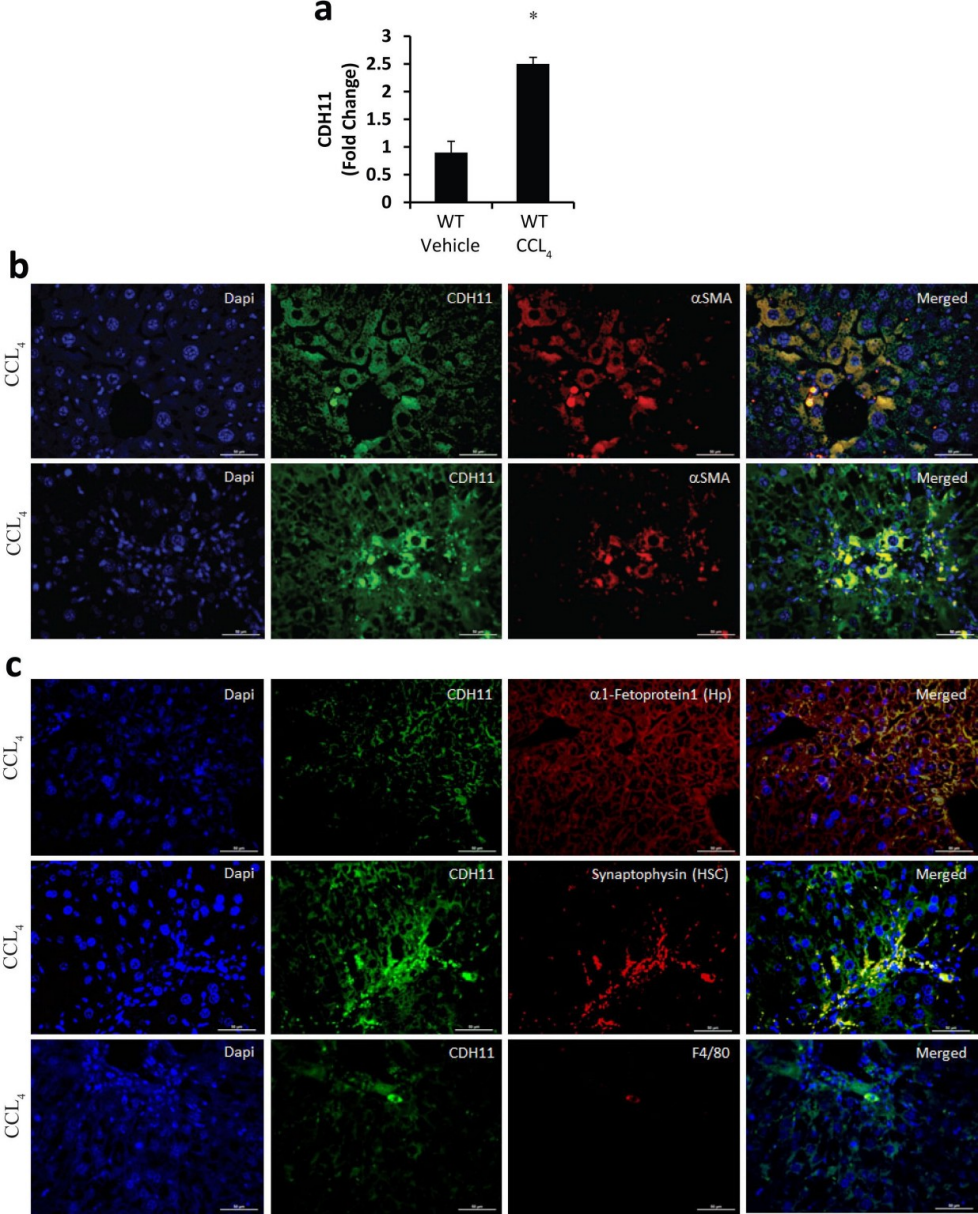
Characterization of CDH11 expression in the CCL_4_ mice model. (a) Liver samples from WT mice treated with corn oil (vehicle) and CCL_4_ were used to isolate total RNA and transcripts were determined for *CDH11*. Transcripts were measured in parallel with 18S rRNA and values are presented as mean of fold change transcripts ± SEM, n≥10 (*, p ≤ 0.05 Vehicle vs. CCL_4_). (b, c) Liver sections from WT mice treated with CCL_4_ were used for co-immunolocalization expression of CDH11 expression (green) and cell type-specific markers for myofibroblasts (b, red, α-SMA) in the central vein (b, top) and sinusoid region (b, bottom), hepatocytes (c, top red, α1-fetoprotein), HSC (c, middle red, synaptophysin) and macrophages (c, bottom red, F4/80). Images are representative of 14 mice from each group. Scale bars: 50 μm.

To determine if CDH11 expression is increased in the CCL_4_ induced liver fibrosis model, total RNA was isolated from livers of WT mice treated with CCL_4_ or vehicle for relative qRTPCR analysis. Compared to vehicle, livers from CCL_4_ treated mice have increased CDH11 mRNA levels (Fig 1A). The increased CDH11 was confirmed by IF where more CDH11 expression was detected in livers from CCL_4_ compared to vehicle. Multicolor IF was performed using cell specific markers to identify the cells expressing CDH11 (α-SMA for myofibroblasts, α1-fetoprotein for hepatocytes, synaptophysin for HSCs, and F4/80 for macrophages). WT mice treated with vehicle did not demonstrate significant levels of CDH11 expression (S1 and S2 Figs). However, in CCL_4_ treated mice, increased CDH11 expression was observed in fibrotic livers localizing to hepatocytes, HSCs and macrophages (Fig 1C). Furthermore, CDH11 expression was also observed on myofibroblasts, co-expressing α-SMA, in the centrilobular area, portal trials and sinusoidal regions (Fig 1B) These data demonstrate that CDH11 is increased in fibrotic livers from CCL_4_ treated mice, localizing to injured hepatocytes, HSCs, macrophages, and myofibroblasts.

**Fig 2:**
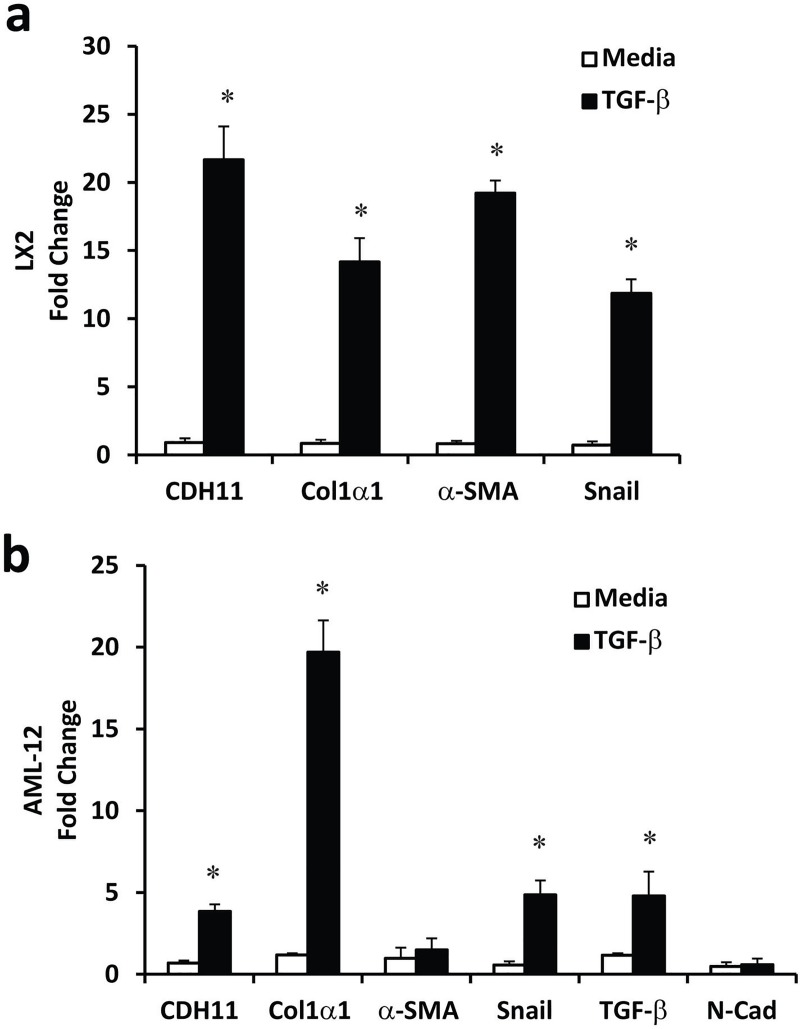
CDH11 expression on hepatic stellate cells and hepatocytes. Total RNA was isolated from LX2 cells, a human hepatic stellate cell line, stimulated with media or TGF-β. TGF-β upregulated expression of *CDH11*, *Col1-*α*1*, α*-SMA*, *and Snail*. Total RNA was isolated from AML-12 cells, a mouse hepatocyte cell line, stimulated with media or TGF-β (b). TGF-β upregulated expression of *CDH11*, *Col1-*α*1*, *TGF-*β, *and Snail*, but not α*-SMA*, or *N-cadherin*. Values are presented as mean of fold change transcripts + SEM, 4 independent experiments were performed in triplicate (*, p ≤ 0.05 Media vs.TGF-β).

To further confirm the expression of CDH11 on hepatic stellate cells and hepatocytes, in vitro cell culture studies were performed. As seen in Fig 2A, the human hepatic stellate cell, LX2, stimulated with TGF-β increases expression of multiple mesenchymal genes, including *Col1-*α*1*, α*-SMA* and *Snail*. Expression of *CDH11* at baseline was low but markedly increased with TGF-β stimulation. In addition, stimulation of AML12 cells, a murine hepatocyte cell line, with TGF-β also increased *Col1-*α*1*, *Snail* and *TGF-*β, but did not markedly increase expression of α*-SMA* (Fig 2B). Interestingly, TGF-β stimulation of AML12 cells upregulated expression of *CDH11* but not N-cadherin. These data provide additional support for the upregulation of CDH11 on hepatic stellate cells and hepatocytes in the fibrotic milieu.

### CDH11 deficiency attenuates liver fibrosis

**Fig 3:**
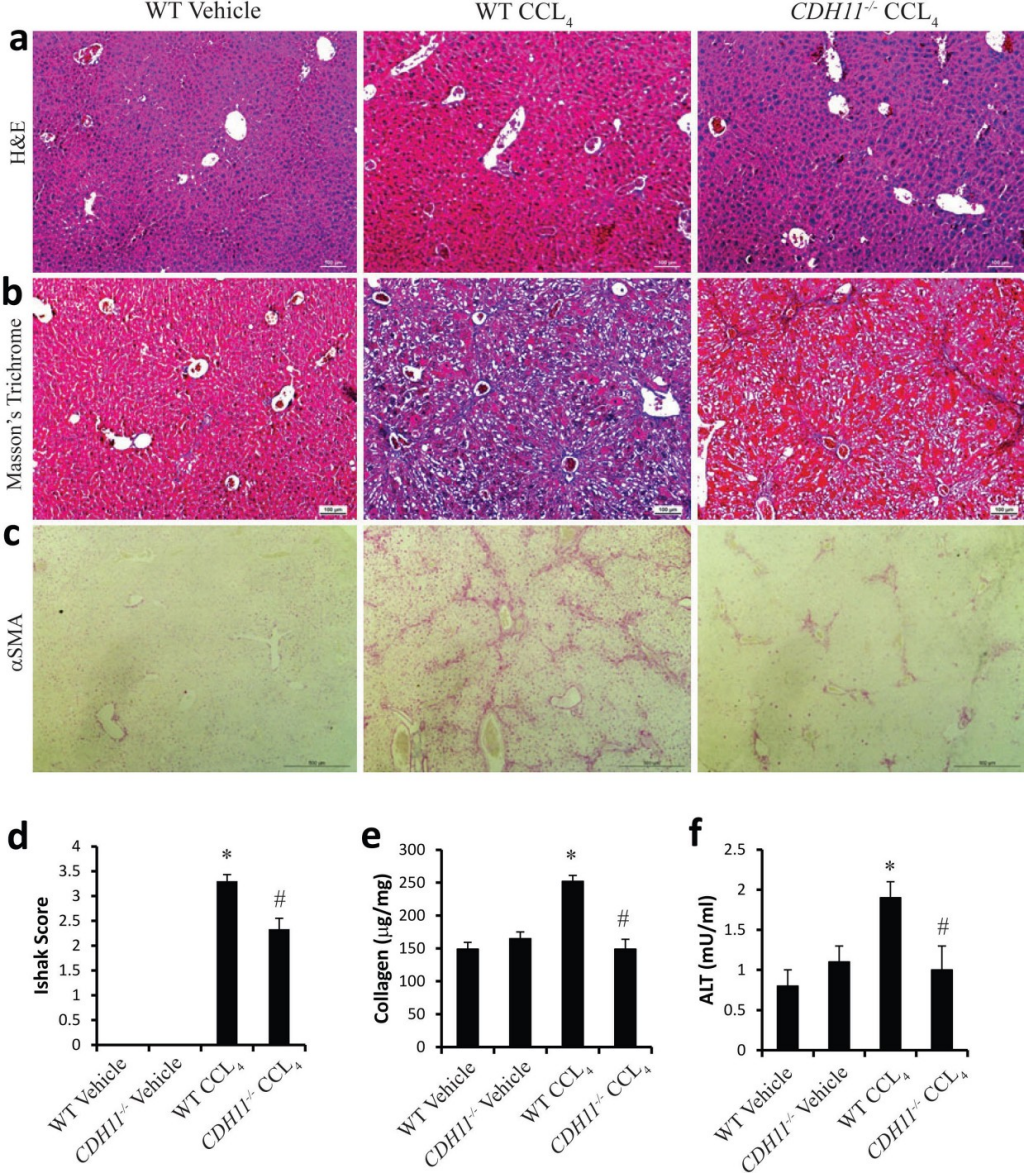
CDH11 deficient mice have attenuated liver fibrosis. Examination of liver histology using (a) H&E staining, (b) Mason’s Trichome, and (c) Col1-α1 from vehicle treated WT mice (left), CCL_4_ treated WT mice (middle), and treated with CCL_4_ treated CDH11^*-/-*^ mice (right). CDH11 deficiency displayed a reduction in liver fibrosis. Images are representative of 14 mice from each group. Scale bars: 100 μm (a, b); 500 μm (c). Compared to WT, CDH11^*-/-*^ mice treated with CCL_4_ have lower (d) histological scores of fibrosis as assessed by Ishak scoring, (e) soluble collagen levels by Sircol, and (f) serum ALT. Data are given as mean +/- SEM, with a total of 14 mice per group performed in 4 independent experiments. (*, p ≤ 0.05 WT Vehicle vs. WT CCL_4_; #, p ≤ 0.05 WT CCL_4_ vs. *CDH11*^*-/-*^ CCL_4_).

To determine the extent to which deficiency of CDH11 attenuates liver fibrosis *in vivo*, CCL_4_ was administered to WT and *CDH11*^*-/-*^ mice for eight weeks. Liver sections from WT mice injected with CCL_4_ demonstrated characteristic centrilobular damage and fibrosis (Fig 3A), increased ECM deposition (Masson’s trichrome staining, Fig 3B), and increased α-SMA expression (Fig 3C). In contrast, *CDH11*^*-/-*^ mice injected with CCL_4_ had decreased fibrosis in all endpoints assessed. *CDH11*^*-/-*^ mice administered CCL_4_ had attenuated centrilobular damage, diminished ECM deposition, and decreased α-SMA expression compared to WT mice (Fig 3A–3C, right panel). Accordingly, *CDH11*^*-/-*^ mice had significantly less histological evidence of liver fibrosis compared to WT mice using Ishak scoring of H&E stained sections (Fig 3D). In addition, Sircol assay demonstrated increased collagen content in CCL_4_ treated WT mice (relative to vehicle, Fig 3E). However, increased collagen was not observed in livers from *CDH11*^*-/-*^ mice administered CCL_4_ (Fig 3E). Finally, serum ALT levels were increased in WT mice but not in *CDH11*^*-/-*^ mice administered CCL_4_ (Fig 3F). Together these data demonstrate that *CDH11*^*-/-*^ deficient mice have markedly attenuated liver fibrosis compared to WT mice in the CCL_4_ mice model.

### CDH11 deficiency decreases pro-fibrotic mediators

**Fig 4:**
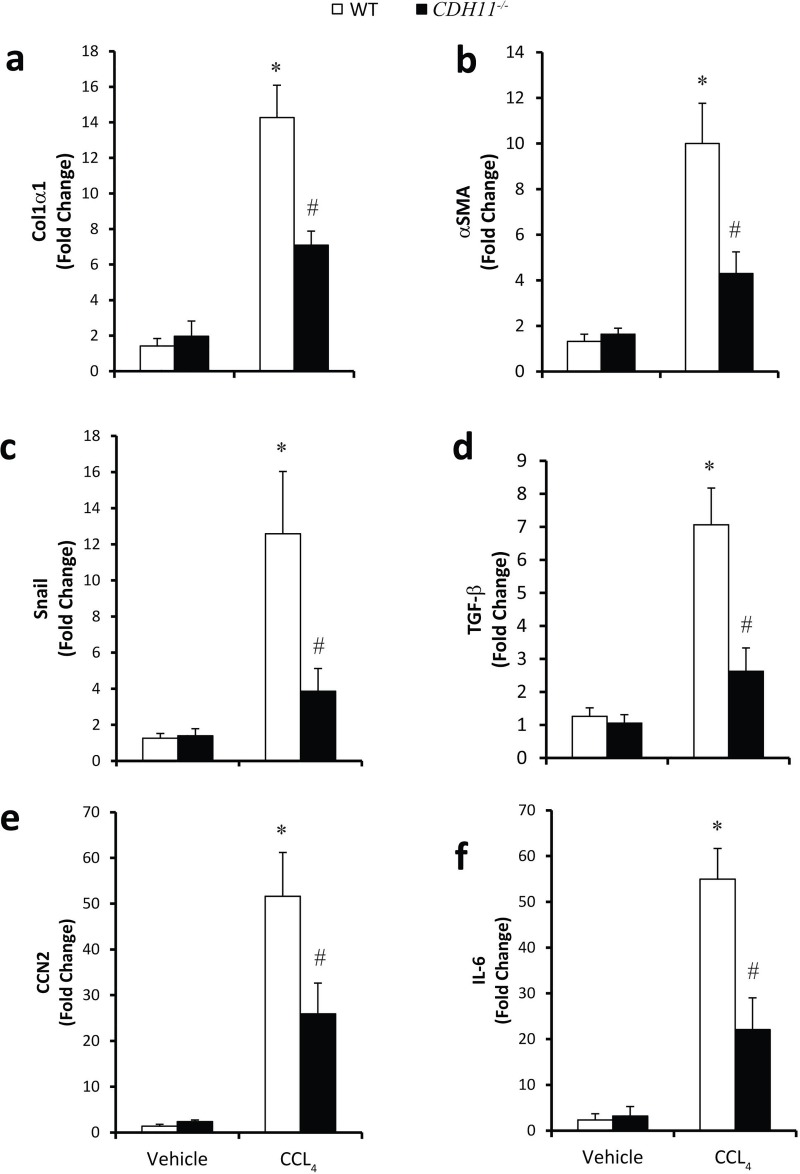
CDH11 deficient mice have decreased expression of fibrotic mediators during development of liver fibrosis. Total RNA was isolated from liver biopsies from these mice and transcripts were determined for (a) *Col1-*α*1*, (b) α*-SMA*, (c) *Snail*, (d) *TGF-*β,(e) *CCN2*, and (f) *IL-6*. Transcripts were measured in parallel with 18S rRNA and values are presented as mean of fold change transcripts. Data are given as mean ± SEM, with a total of 14 mice per group performed in 4 independent experiments, (*, p ≤ 0.05 WT Vehicle vs. WT CCL_4_; #, p ≤ 0.05 WT CCL_4_ vs. *CDH11*^*-/-*^ CCL_4_).

To further quantify the extent of liver fibrosis in WT and *CDH11*^*-/-*^ mice, total RNA was isolated from livers of mice treated with vehicle or CCL_4_ and levels of fibrotic mediators was assessed using relative qRTPCR. As seen in Fig 4 and S3 Fig, liver samples from WT mice treated with CCL_4_ had increased expression of *Col1-*α*1*, α*-SMA*, *Snail*, *TGF-*β, *CCN2*, and *lumican* compared to vehicle. In contrast, the CCL_4_ driven increase in expression of *Col1-*α*1*, α*-SMA*, *Snail*, *TGF-*β, *CCN2*, and *lumican* was attenuated in *CDH11*^*-/-*^ mice. These data demonstrate that CDH mice have less fibrogenesis than WT mice. Additionally, *IL-6*, which has pro-inflammatory and pro-fibrotic properties [9, 12–14], was also increased in WT mice but not in *CDH11*^*-/-*^ mice administered CCL_4_. Finally, to determine if CDH11 also regulated enzymes involved in fibrolysis, the expression of matrix degrading enzymes such as MMP3 and MMP13 and an inhibitor of MMPs, tissue inhibitor of metalloproteinases (TIMP1) was determined. As seen in S3 Fig, levels of MMP3 and MMP13 as well as TIMP1 were increased in fibrotic livers of WT mice, but attenuated in *CDH11*^*-/-*^ mice. Together, these data demonstrate that *CDH11*^*-/-*^ mice have decreased liver fibrosis induced by CCL_4_.

## Discussion

The central hypothesis tested is that CDH11 is an important mediator of liver fibrosis. The data presented herein, demonstrate that CDH11 is elevated in fibrotic liver tissue from CCL_4_ treated mice, localizing to myofibroblasts in the centrilobular area, portal triads, and sinusoid region, injured hepatocytes in the centrilobular region and portal triads, HSCs and macrophages. Furthermore, compared to WT mice, CDH11 deficient mice develop significantly less liver fibrosis when administered CCL_4_. These data demonstrate an important role of CDH11 in the development of liver fibrosis.

CDH11 is an adhesion molecule that is a member of the cadherin superfamily. However, CDH11 likely has functions beyond its role in adhesion [15]. CDH11 has been reported to play an important role in cancer metastasis [16] and inflammatory arthritis [10]. In addition, CDH11 also plays a role in the development of skin and lung fibrosis. CDH11 expression is increased in fibrotic skin of scleroderma patients and mouse models of skin fibrosis [7, 9] and in fibrotic lungs of idiopathic pulmonary fibrosis patients and mouse models of pulmonary fibrosis [8]. The current report now shows that CDH11 expression is also increased in fibrotic livers of mice treated with CCL_4_ and CDH11 modulates the development of liver fibrosis. These data are important as they demonstrate that CDH11 is increased in multiple fibrotic organs and its expression can be increased by stimuli other than bleomycin. These data implicate CDH11 as a common mediator of fibrosis across multiple tissues and suggest that targeting CDH11 therapeutically may have very broad implications for a number of fibrotic diseases.

The mechanisms of how CDH11 regulates liver fibrosis were not addressed in this report. However, the expression of CDH11 in multiple key cellular populations suggest that CDH11 likely regulates multiple steps in the development of fibrosis. For example, the expression of CDH11 on the myofibroblasts suggests that it may regulate the production of ECM. Indeed, CDH11 has been shown to regulate dermal fibroblast and aortic smooth muscle cell production of ECM components, such as collagen and elastin [17]. In dermal fibroblasts, CDH11 deficiency leads to decreased collagen synthesis whereas CDH11 engagement through homophillic interactions led to increased collagen production via ROCK and TGF-β pathway activation and subsequent MYPT and SMAD2 phosphorylation [17].

However the expression of CDH11 on other cells, including injured hepatocytes, HSCs and macrophages, during the development of liver fibrosis suggests roles for CDH11 beyond that of regulating myofibroblasts. First, pro-fibrotic cytokines, such as TGF-β, are secreted by multiple cells, including macrophages, and play an important role in the differentiation of fibroblasts to myofibroblasts. Indeed CDH11 has been show to regulate TGF-β production in macrophages [7, 9]. A recent report has confirmed the expression of CDH11 on macrophages and suggested it may promote lung fibrosis through CDH11 mediated contact with fibroblasts [18]. Second, CCL_4_ administration to mice induces hepatocyte injury and apoptosis [19]. Hepatocyte injury leads to the activation of Kupffer cells and HSCs, which results in increased TGF-β production and myofibroblast differentiation [20]. Indeed, the current data suggest that CDH11 is upregulated during hepatocyte injury, similar to prior observations of CDH11 on injured type II alveolar epithelial cells in IPF patients and murine bleomycin lung fibrosis [8, 9]. Therefore, it is possible that CDH11 regulates the cellular response to injury. Third, during the development of liver fibrosis, HSCs are considered a major source of myofibroblasts. The upregulation of CDH11 on HSC may contribute to HSC activation. Interestingly, besides HSCs, hepatocyte EMT may also contribute to the myofibroblasts accumulation, in the liver [21]. Hepatocyte EMT has been demonstrated *in vivo* using transgenic mice, in which hepatocyte-derived cells are labeled with β-galactosidase (β-gal). Treating these mice with CCL_4_ resulted in 45% of the cells co-expressing the mesenchymal marker, fibroblast-specific protein 1, and β-gal. CDH11 has been shown to regulate EMT in alveolar epithelial cells [8] and cancer cells, therefore, the expression of CDH11 on HSC and injured hepatocytes suggests that CDH11 may regulate EMT during liver fibrosis. Therefore, CDH11 may be a central player in the development of liver fibrosis through regulation of multiple key cells in fibrogenesis including hepatocytes, HSC, macrophages and myofibroblasts.

In conclusion, the current manuscript highlights the importance of CDH11 in the development of liver fibrosis. We hypothesize that hepatic injury by CCL4 increases CDH11 expression on a number of important cells, such as the injured hepatocytes, macrophages, HSCs, and myofibroblasts. The upregulation of CDH11 expression on these cells helps promote the pro-fibrotic environment by increasing mesenchymal gene expression and the deposition of ECM in tissues. These data implicate CDH11 as a mediator of liver fibrosis, adding to the growing body of evidence that CDH11 is a common mediator of fibrosis in multiple tissues and suggest that targeting CDH11 is an intriguing therapeutic strategy for fibrotic disease including liver fibrosis.

## Supporting information

S1 Fig Characterization of cadherin-11 expression in wild type mice. (a) Isotype conjugated antibodies (alexa 488; alexa 647) were used in liver biopsies from wild type mice (WT) treated with CCL_4_. (b) Co-immunolocalization expression of CDH11 expression (green fluorescence) and cell type-specific markers (α1-Fetoprotein for hepatocytes) was determined in liver sections from wild type mice treated with vehicle. Images are representative of 14 mice from each group. Scale bars: 50 μm.
(TIF)

S2 Fig Co-localization of cadherin-11 expression in wild type mice. Liver sections from wild type mice treated with vehicle were used for co-immunolocalization expression of CDH11 expression (alexa 488, green) and cell type-specific markers (α-SMA for myofibroblasts; synaptophysin for hepatic stellate cells; and F4/80 for macrophages; alexa 647 red). Images are representative of 14 mice from each group. Scale bars: 50 μm.
(TIF)

S3 Fig Total RNA was isolated from liver biopsies from these mice and transcripts were determined for (a) *TIMP1*, (b) *MMP13*, (c) *MMP3*, and (d) *Lumican*. Transcripts were measured in parallel with 18S rRNA and values are presented as mean of fold change transcripts. Data are given as mean ± SEM, with a total of 14 mice per group, (*, p ≤ 0.05 WT Vehicle vs. WT CCL_4_; #, p ≤ 0.05 WT CCL_4_ vs. *CDH11*^*-/-*^ CCL_4_).
(TIF)

S1 File Animal research; reporting of *in vivo* experiments guidelines. (PDF)

S2 File Raw numerical data. (XLSX)
